# The usefulness of pedometry in patients with chronic obstructive pulmonary disease

**DOI:** 10.1186/2049-6958-8-7

**Published:** 2013-02-05

**Authors:** Nicoleta Bertici, Ovidiu Fira-Mlădinescu, Cristian Oancea, Voicu Tudorache

**Affiliations:** 1 Department of Pneumology, University of Medicine and Pharmacy Victor Babe, Timisoara, Romania

**Keywords:** COPD, Daily physical activity, Effort tolerance, Pulmonary rehabilitation, Quality of life

## Abstract

**Background:**

Effort tolerance and daily physical activity (DPA) are predictive of quality of life and survival in COPD patients, but still remain difficult to assess based on their daily life.

The aim of this study was: how to relate pedometry to other classic parameters commonly used in pulmonary rehabilitation (PR).

**Methods:**

DPA was evaluated through pedometry. 74 patients with COPD, aged 63.55 ± 8.73 (12 stage II, FEV_1_ = 60.16 ± 7.78%), (29 stage III, FEV_1_ = 39.07 ± 6.30%), (33 stage IV, FEV_1_ = 23.1 ± 7.18%). The monitoring was conducted for a period of 7 days before and 6 months after a pulmonary rehabilitation program (PRP) of 3 weeks. A control group consisting of 21 patients with stable COPD was evaluated initially, but they did not undergo Pulmonary Rehabilitation Program (PRP). After 6 months the patients were re-evaluated using the same parameters.

**Results and discussion:**

The values are widely dispersed, with a maximum of 17,420 and minimum of 964 steps/24hrs. The average values acquired were: the lowest in COPD stage IV (2476→3112 steps/24 hrs, p < 0.0001), still with the highest increase over 6 months of PR + 636steps/24hrs; in COPD stage III the increase of DPA was + 597steps/24hrs over 6 months (5627→6224, p < 0.0001), COPD stage II registered the lowest increase + 540steps/24hrs (8724→9264, p < 0.13), probably because the subjects belonging to this stage had the best preserved DPA. The results show moderate correlation between pedometry and the 6MWT and the SGQ. (r = 0.5-0.7). However it demonstrated the positive effects of PRP, even after 6 months.

**Conclusions:**

DPA decreases with increasing COPD stage, it is fluctuant with every subject, dependent on clinical status, weather and daily schedule. Wearing pedometers is very easy and motivational, provided that patients realize that they are being “watched”.

## Background

The current definition of COPD stipulates that this is a complex syndrome with significant extrapulmonary manifestations [1], where individuals evaluation is both useful and necessary. The heterogeneity lies in the clinical and physiological manifestations, the treatment response, the deterioration of respiratory function and survival [2], which has led to the initiation of future perspectives of phenotype of COPD. These are done through distinct clinical, therapeutic, and prognostic subcategories. Amongst the significant number of extrapulmonary determinations, the loss of muscular mass induced by the physical inactivity due to dyspnoea is very important, as it determines the decrease of effort tolerance and life quality.

Daily physical activity (DPA) is considered as the total of voluntary movements produced by skeletal muscles above resting levels, carried out during the day [3]. A study made by Watz et al. [4]. showed that there is a gradual reduction in the DPA level in COPD patients. Effort tolerance and DPA are predictive of life quality and survival in COPD patients, but still remain difficult to assess based on their daily life [5,6].

Pedometers were initially used in sports. They also proved their usefulness in the medical field [7,8]. They register the physical activity (number of steps done) and estimate the distance in meters and the number of kilocalories burned. The values are recorded and allow comparison of results. It is recommended the pedometer be worn daily at the belt and maintained during the whole day. 10,000 steps are recommended per day. These are the equivalent of 8 km as a standard for an active and healthy life [9].

### Aim

To evaluate the utility of DPA monitoring through pedometry in COPD patients and to relate pedometry with other classic parameters commonly used in pulmonary rehabilitation (PR), such as the 6-minute walking test (6MWT) and Saint-George Quality of Life Questionnaire (SGQ).

## Methods

DPA has been evaluated by pedometry in the case of 74 patients (men) with stable COPD, without significant associated pathology and without physical limitations, aged 63.55±8.73 (12 stage II, FEV_1_ =60.16±7.78%), (29 stage III, FEV_1_=39.07±6.30%), (33 stage IV, FEV_1_ =23.1±7.18%). These patients were monitored for a period of 7 days. The test was done before and after 6 months of Pulmonary Rehabilitation Programs (PRP) of 3 weeks (Tables 1 and 2).

**Table 1:** Characteristics of the three groups of patients COPD

		**COPD II**	**COPD III**	**COPD IV**
Patients numbers		12	29	33
Mean Age 63.55 ± 8.73		59.33 ± 4,59 4.59	62.44 ± 10,36 10.36	65,77 ± 7,66 7.66
FEV_1_ (%)		60.16 ± 7.78	39.07 ± 6.30	23.1 ± 7.18
Origin	Urban	66.5% (8 patients)	51.7% (15 patients)	48.48% (16 patients)
			52.7% (39 patients)	
	Rural	33.3% (4 patients)	48.2% (14 patients)	51.5% (17 patients)
			47.2% (35 patients)	
Body Mass Index	Normal	66.6% (8 patients)	44.8% (13 patients)	36.3% (12 patients)
			44.5% (33 patients)	
	Denutrice Wasted	8.33% (1 patient)	34.4% (10 patients)	33.3% (11 patients)
			29.7% (22 patients)	
	Obesity	25% (3 patients)	20.6% (6 patients)	30.3% (10 patients)
			25.6% (19 patients)	

**Table 2:** Results of monitoring the daily physical activity by pedometry

***Group***	***N. patients***	***Initially***	***SD***	***After 6 month***	***SD***	***Change***	***p***
COPD II	12	8724.33	2908.34	9264.16	2405.18	+ 540	0.13
COPD III	29	5627.44	2152.95	6224.42	2105.19	+ 597	0.0001
COPD IV	33	2476.32	2104.12	3112.63	2088.46	+ 636	0.0001

We split our patients in this group after spirometry, taking into consideration the GOLD guideline 2009. Initially, each patient had to do spirometry, 6-MWT and SGQ. Then they had to wear a Canyon pedometer during the entire day, every day for a week and had to note the numbers of steps/24 hours and some comments. The week log contains patient data (name, age, gender), weight, height, body mass index, profession, origin (urban or rural), starting day (season), steps/day and comments on physical training, spending leisure time and weather. They all had to undertake a PRP for three weeks. Included in the program were the following aims: understanding the disease, helping patients to give up smoking, cough and sputum education, adapted diet and physical training with the purpose of stimulating the respiratory and periferic muscles. The rehab program was carried out in the Department of Pulmonary Rehabilitation of the Victor Babes Clinical Hospital of Timisoara on inpatients for 3 weeks (the duration was approved by the Romanian Health Authorities). Patients followed sessions of daily training 5 day/week under the supervision of physiokinotherapists. The physical effort was individualized for each patient, according to the severity of the disease, age, BMI, and the pre-existent physical condition. At the beginning the duration and the intensity of the effort was low (15–20 minutes of the 70% of the functional capacity). These were increased every 2–3 days. In association there were film sessions, leaflets with medical education with information concerning the disease,treatment, and manner of administration, diet, risks (held by pulmonologists, psychologists, nurses in working day).

After six months, patients were re-evaluated under the same circumstances with 6-MWT, SGQ, and pedometry (Tables 3 and 4).

**Table 3:** The results obtained to monitoring effort tolerance (6 MWT) and quality of life (SGQ)

**COPD stage (N. patients)**	**6 Minutes walking test (meters)**			**Quality of life (SGQ -%)**		
	**Initially**	**After 6 months**	**Changes**	**Initially**	**After 6 months**	**Changes**
**COPD II (12)**	472.50±63.29	524.58± 62,28	**+52**	42.47±5.98	38.08±5.20	**−4.3**
**COPD III(29)**	415.03±100.0	456.31±109.0	**+41**	68.17±10.52	62.81±9.549	**−5.3**
**COPD IV(33)**	350.20±83.76	383.36±86.75	**+33**	75,33±12.60	68.58±11.82	**−6.7**

The control group of 21 patients with COPD that did not follow the PRP, was evaluated under similar conditions. An increase in DPA of + 52.14 steps/day was noticed without being statistically significant (p = 0.25).

**Table 4:** The results obtained to monitoring effort tolerance (6MWT), quality of life (SGQ) and pedometry in control group

**Control group**	**Patients number**	**6MWT Initial (meters)**	**6MWT after 6 months (meters)**	**QL initial (%)**	**QL after 6 months (%)**	**Pedometry initial (steps/day)**	**Pedometry after 6 months (steps/day)**
COPD II	5	487.6	485.2	43.14	44.12	8620.8	8672.2
COPD III	7	436.4	431.57	61.87	63.35	5743.4	5810.14
COPD IV	9	349.5	342.22	69.48	69.73	2551.1	2592.4
Total/Change	21	- 4.15		+ 0.72		+ 52.14 (p = 0.25)	

When released from hospital, each patient received a list with recommendations regarding physical activity (daily gymnastics, minutes to walk/run, fitness), and diet. Afterwards, a contact with the patients was made by telephone every two weeks. The nurses questioned the patients about their adherence to each chart, and encouraged them to continue the program. We wanted to stimulate them to continue their physical training and treatment as recommended.

A control group consisting of 21 patients with stable COPD (5 stage II, 7 stage III, and 9 stage IV) were evaluated initially, but they did not undergo PRP. After 6 months, they were re-evaluated using the same parameters (Table 5).

**Table 5:** The results obtained to monitoring the daily physical activity by pedometry in control group

**Control group**	**No. of patients**	**Initially**	**SD**	**After 6 month**	**SD**	**Change**	***p***
COPD II	5	8620.8	1734	8672.2	1607	+ 51.2	0.64
COPD III	7	5743.4	875.5	5810.1	852.5	+ 66.7	0.34
COPD IV	**9**	2551.1	653.6	2592.4	749.7	+ 41.3	0,61

The analysis of the results was performed by calculating the average value (VM) and the standard deviation (SD) to each group and for each parameter monitored. The Pearson (high statistical value p <0.05) test was used to establish the level of the statistically significant modifications that occurred after 6 months, and with the Beaghehole index 1997 (r<0.20 absent correlation and r≥0.70 strong correlation) the association/correlation strength of these parameters was decided upon. To evaluate the evolution of each patient we used the paired *t* test. To evaluate the normality of dispersion of the studied groups we used D’ Agostino test.

## Results and discussion

Out of the 103 patients initially evaluated, only 74 completed the re-examination after 6 months. There were 29 patients that dropped out (5 had exacerbations, with 8 we could not keep in touch, 8 had forgotten to use pedometers daily for a week, 3 had forgotten and mistaken data, 1 lost the agenda with the data, and 4 refused the 6 month evaluation). The values registered by pedometry were widely dispersed with a maximum of 17,420 and minimum of 964 steps/24 hours. The best preserved DPA had COPD II. The highest increase over 6 months of PR had COPD IV. Only 33 patients had a normal BMI (44.59%). The others were underweight (29.7%/22 patients) and overweight (25.6%/19 patients).

The results showed high correlation between 6MWT and SGQ, whereas between pedometry and the two above tests the correlation was moderate (r = 0.5 – 0.7).

Patients from an urban background have prevailed because of greater addressability, higher education level and better compliance to PRP. As expected, there was a greater physical activity to patients with occupations that imply physical work, and to those from rural areas due to harder labour. The high level of dispersion of the inter-individual pedometry value is showed by the high standard deviation for each group.

As demonstrated by other studies, [4,6] DPA decreased with increasing severity of COPD. The significant improvement recorded after PRP is noteworthy, but only in severe stages (III and IV), probably because these patients have the most severe restriction and are most compliant to treatment and medical advice, while in early stages patients still have largely preserved exercise tolerance.

The patients with extreme values of BMI walked less, especially those with cachexia, but the differences were not statistically significant (p-0.15).

A deficiency of the study was that DPA monitoring was conducted during different seasons (every six months), and it has been found that weather influences physical activity, being higher in spring and autumn, lower in summer, and especially lower in winter.

More deficiencies were found in the case of the 6MWT, which has proven to depend on many factors (temperature and humidity, clothing and footwear worn, food, time of the test, medication, patients’ psycho-emotional status and level of motivation/stimulation during the test).

The various elements that influence both 6MWT and the physical activity evaluated by pedometers explain the low degree of correlation between the results provided by these parameters.

In comparison to the 6MWT (often considered tiresome and stressful), pedometers were much better received by the patients, being labelled as “personal toys” that follow them and force them to be more active. The only problem was that there were patients that forgot to put them on in the morning. At the end of the study, there were some patients who were interested in buying such a device.

This study also proved PRP efficiency. This is similar to other reports [10-12] by emphasizing the significant improvement of DPA, 6MWT, and QL I in the case of monitored patients. The effects were long-term, maintained to 6 months.

## Conclusions

DPA decreased with increasing COPD stage. It is fluctuant from subject to subject, depending on the clinical status, BMI, weather/season, profession and daily schedule. Wearing pedometers is very easy and motivational, provided that patients realize that they are being “watched”. All these factors determine the PA level. This is the reason why both its objective evaluation and the establishment of reference data for each COPD stage are very difficult. But, at an individual level, these simple instruments proved their utility, allowing self-monitoring. We suggest that a 10% decrease in average DPA should be taken as an alarming signal for the outcome of these patients (possible complications or exacerbations).

## Abbreviations

6MWT: Six Minutes Walking Test; BMI: Body Mass Index; COPD: chronic obstructive pulmonary disease; DPA: daily physical activity; FEV_1_: forced expiratory volume in 1 second; GOLD: Global Initiative for Chronic Obstructive Lung Disease (2009); PRP: pulmonary rehabilitation programs; SGQ: Saint George’s Respiratory Questionnaire of Quality of Life; SD: standard deviation; VM: values means.

## Competing interests

The authors declare that they have no competing interests.

## Authors' contributions

NB conceived the study, participated in its design, coordinated, and helped to draft the manuscript; OFM performed the statistical analysis; CO coordinated the development of the Pulmonary Rehabilitation Program; VT supervised the development of the study. All authors read and approved the final manuscript.
